# Cardiac Deformation Patterns During Exercise in Healthy Children

**DOI:** 10.3390/jcdd12120488

**Published:** 2025-12-10

**Authors:** Dario Collia, Ling Li, Mary Craft, Christopher C. Erickson, Zahi A. Fayad, Maria G. Trivieri, Jason Christensen, Gianni Pedrizzetti

**Affiliations:** 1 Department of Engineering and Architecture, University of Trieste, 34127 Trieste, Italy; 2 Department of Cardiovascular Surgery, Icahn School of Medicine at Mount Sinai, New York, NY 10029, USA; 3 BioMedical Engineering and Imaging Institute, Icahn School of Medicine at Mount Sinai, New York, NY 10029, USA; 4 University of Nebraska Medical Center, Children’s Nebraska, Omaha, NE 68114, USA; 5 Cardiovascular Institute, Icahn School of Medicine at Mount Sinai, New York, NY 10029, USA

**Keywords:** strain, CFD, hemodynamic forces, physical exercise, children cardiovascular, blood flow, vorticity

## Abstract

In the cardiovascular system, geometric remodeling of the cardiac chambers is the main mechanism enabling increased cardiac performance during exercise in athletes, as well as underlying pathological progression toward heart failure. In this study, we investigated cardiac mechanics in healthy children across five phases of physical exercise, *Rest*, *Mid*, *Peak*, and *Recovery*, at 5 and 10 min, using three-dimensional echocardiography. Analyses were conducted relative to a reference cohort of healthy children to identify exercise-induced modifications that may contribute to cardiac remodeling. Ventricular performance was assessed through two complementary approaches: myocardial deformation, quantified by the principal values and directions of the strain tensor, and intraventricular flow dynamics, including assessments of ventricular filling patterns as the vorticity, vortex formation time and hemodynamic forces. This preliminary study offers promising insights into early cardiac function changes that may inform our understanding of cardiac remodeling during adaptation, healing or disease progression.

## 1. Introduction

Progressive modifications in cardiac geometry are accompanied by corresponding changes in functional parameters, a process collectively known as cardiac remodeling. Although commonly associated with pathological conditions arising as a consequence of various cardiomyopathies, which may lead to heart failure [1,2] the remodeling mechanism can also occur as a beneficial adaptive response to physiological stimuli such as physical exercise. This favorable adaptation is characterized by structural and functional enhancements that improve cardiac performance to meet elevated circulatory demands, as exemplified by the physiological remodeling seen in the athlete’s heart [3]. However, knowledge of the physiological changes in pediatric athletes driven by growth, maturation, and training remains incomplete, despite growing research interest over the past decade [4]. While the cardiovascular adaptations to athletic training are well documented in adults, corresponding data in children, particularly those in early stages of athletic development, are limited [5,6,7]. Elucidating these mechanisms in the pediatric population is essential for distinguishing normal adaptive remodeling from potential early markers of maladaptation.

Understanding the mechanisms underlying both pathological and physiological forms of cardiac remodeling is of paramount importance across various domains of cardiology. It informs efforts to predict disease progression, assess physical exercise recovery, monitor athletic performance, and even track cardiac development in growing children. Despite its clinical relevance, current models of cardiac remodeling remain limited and often lack robust predictive power [8].

Exercise stress echocardiography is established as an important tool for dynamic cardiac evaluation and has been shown to have prognostic value in diverse clinical conditions, including left bundle branch block, coronary artery disease, and atrial fibrillation [9]. Yet, its application for characterizing normal pediatric adaptation during exercise remains underexplored.

In this context, the present study analyzes the functional responses of the left ventricles in healthy children during physical exercise using three-dimensional (3D) echocardiography. LV geometry and function were analyzed across five sequential phases of exercise: *Rest*, *Mid*, *Peak*, *Recovery 5* (5 min post-exercise) and *Recovery 10* (10 min post-exercise).

This analysis integrates both myocardial deformation imaging and image-based fluid dynamics modeling. Strain tensor evaluation derived from 3D echocardiographic sequences quantifies both the magnitude and direction of myocardial contraction, offering a comprehensive characterization of LV deformation patterns throughout the various phases of physical exercise in response to an adaptive effort. Additionally, image-based numerical simulations facilitate the analysis of intraventricular fluid dynamics, including quantification of vorticity, vortex formation time (VFT), and hemodynamic forces (HDF), and provide novel insights into ventricular performance and efficiency during physiological stress.

## 2. Material and Methods

### 2.1. Clinical Data

The study cohort consisted of 19 healthy children (age: 9.7 ± 2.3 yrs, 53% male–47% female, Body Surface Area (BSA): 1.4 ± 0.2 m^2^, Body Mass Index (BMI): 18.1 ± 3.7 Kg/m^2^) all of whom underwent a standardized physical exercise test. An independent age-matched reference group of 25 healthy children (age: 10.6 ± 3.4 yrs, 60% male–40 female%, BSA: 1.2 ± 0.3 m^2^, BMI: 17.9 ± 3.1 Kg/m^2^) was used for comparison. All participants demonstrated normal cardiac anatomy and function on screening and had no known medical conditions. All subjects underwent 3D echocardiography following a non-invasive protocol adopted at collaborating clinical institutions for screening purposes. Clinical data for the study cohort are summarized in Table 1.

### 2.2. Geometry

**Figure 1:**
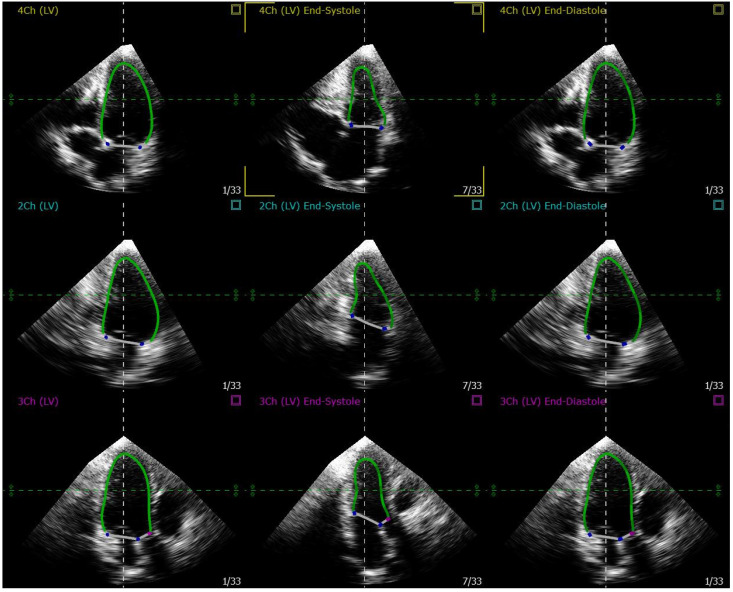
Graphical user interface of an example of endocardial border tracing at end-systole and end-diastole using the semi-automatic softwareTomTec (4D LV-Analysis and 4D MV-Assessment).

3D echocardiography acquisitions were performed with spatially focused imaging centered on the LV to ensure high temporal resolution. The acquired 3D datasets were analyzed at the same clinical institutions using dedicated software (4D LV-Analysis, TomTec Imaging System GmbH, Unterschleissheim, Germany) to extract dynamic ventricular geometry. The endocardial border -the interface between myocardial tissue and blood pool- was semi-automatically traced at end-systole and diastole via the graphical user interface as shown in Figure 1.

**Figure 2:**
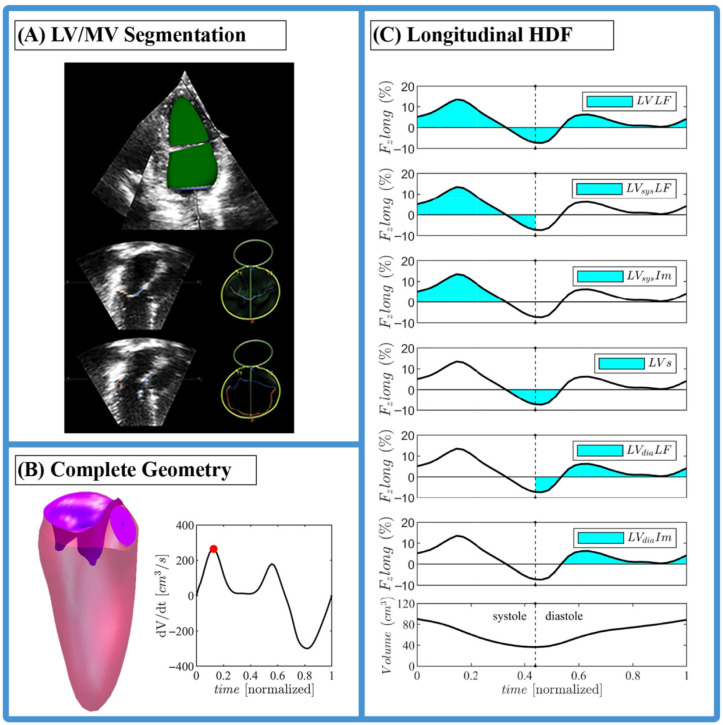
(**A**) 3D LV and MV segmentation procedure with TomTec software (4D LV-Analysis and 4D MV-Assessment). (**B**) Complete geometry of one of the control cases in semi-open MV configuration. (**C**) Graphic description of the longitudinal diastolic and systolic hemodynamic force parameters.

The software then applies an optical flow algorithm to track anatomical features across the cardiac cycle. This process produced a triangulated surface representation of the moving endocardial geometry, where each vertex corresponds to a material point (Figure 2A). The mitral valve (MV) geometry, located at the LV inlet, was extracted using companion software (4D MV-Assessment; TomTec Imaging Systems GmbH, Unterschleissheim, Germany). An example of the 3D segmentation process for both LV and MV using the mentioned software is shown in Figure 2A.

The key advantage of 3D echocardiography is its ability to reconstruct a sufficient number of frames for dynamic functional analysis. In this study, the average number of frames per minute was: Control cohort = 32±5; study cohort: Rest = 26±7, Mid = 12±1, Peak = 11±1, Recovery5 = 17±2 and Recovery10 = 19±4. As exercise intensity increased, the number of captured frames decreased due to the limited capacity of 3D echocardiography to track the progressively higher velocities of the endocardial wall. Increasing the frame rate is not feasible, as it can cause stitching errors and gating artifacts [10], which compromise motion detection. These artifacts are more common at higher frame rates [11], particularly during the *Mid* and *Peak* exercise phases. Nevertheless, all recordings maintained ≥10 frames per cardiac cycle, ensuring reliable wall-motion analysis.

To enable group-level comparison, LV geometries were spatially registered and averaged within each cohort. Each mode was translated to a common geometric center and rotated in 3D to align along a consistent anatomical axis-defined as the vector extending from the midpoint between the atrioventricular valve annulus center to the ventricular apex. This alignment ensures uniform valvular positioning across all subjects. Since all participants exhibited normal MV anatomy with comparable characteristics, consistent with prior literature [12], a single representative MV geometry was used for averaged LV reconstructions to simplify and standardize comparison.

This methodology enables the reconstruction of structurally complete LV geometry by integrating the mitral plane (segmented using TomTec 4D LV-Analysis) with the MV (segmented using TomTec 4D MV-Assessment). The MV is subsequently attached to the mitral plane of the ventricle during post-processing using MATLAB software (R2024b, MathWorks, Natick, MA, USA), ensuring alignment in both position and orientation between the MV and LV geometries [13]. The aortic valve (AV), located at the LV outlet, was then modeled using a simplified binary configuration (fully open or fully closed). Its spatial positioning within the LV geometry was inherently determined by the registration process, requiring no further manual alignment procedure. A complete 3D geometry example, including both valves, is shown in Figure 2B.

### 2.3. Exercise Echocardiography

All study subjects underwent semi-supine exercise to exhaustion, followed by a 3-min cool-down period. Echocardiographic data were acquired at five time points: *Rest*, baseline before exercise initiation; Mid-exercise (*Mid*), corresponding to exercise at a heart rate (HR) midway between baseline and the target HR, calculated as [(220-subject age) × 0.85-baseline HR]/2 + baseline HR beats/min; *Peak*, defined as exercise at a target HR of (220-subject age) × 0.85 beats/min; *Recovery 5*, five minutes post peak exercise; *Recovery 10*, ten minutes post peak exercise. The control reference group was recorded only at the *Rest* phase to provide a reference.

All recordings were initially analyzed on an individual basis. Changes in cardiac function during physical exercise, as well as the comparisons with the control group, were statistically assessed using global functional parameters, including ventricular volumes, ejection fraction (EF), and myocardial strain. To enhance spatial comparisons of deformation patterns across different exercise phases, LV geometries were averaged within each group at each time point. This averaging procedure involved translating each ventricular model to a common geometric center, identifying key anatomical landmarks (the mitral annulus, the center of the aortic outlet, and the apex), and applying 3D rotational alignment to standardize ventricular orientation and ensure consistent valvular positioning across all datasets.

### 2.4. Fluid Dynamics

Intraventricular fluid dynamics were evaluated using numerical solutions of the Navier–Stokes and continuity equations. The computational framework, previously described and validated for cardiac flow applications in earlier methodological studies [13,14], is briefly summarized here. The simulations employ an immersed boundary method (IBM), with time integration carried out using a fractional step method in combination with a third-order Runge–Kutta explicit scheme. No-slip boundary conditions are applied on the moving immersed boundaries, which comprise the ventricle geometry and valve surfaces. The motion of the LV boundary was directly imposed using geometry extracted from clinical imaging. For the MV leaflets, movement is obtained using a kinematic condition that enforces the leaflet surface to move at the same velocity as the surrounding fluid at each point. The aortic valve (AV) is modeled as a simplified orifice with a flat surface that toggles between fully open and fully closed states. The AV is considered open when the MV is closed, and during this phase, the normal velocity, averaged over the AV surface, is directed outwards.

### 2.5. Hemodynamic Forces

The dynamic interactions between blood flow and surrounding myocardial tissues have special relevance in ventricular function. Flow-driven forces, in particular, have been shown to influence predominant direction in a healthy ventricle. The computation of HDFs, detailed in previous studies [15], is briefly summarized below and is based on the following formulation:

(1) $$F\left(t\right)=\rho \int_{S(t)}\left[x\left(\frac{\partial v}{\partial t}\cdot n\right)+v\left(v\cdot n\right)\right]dS;$$

where ρ is the fluid density, x the 3D space coordinate, v the fluid velocity field and n the outward unit normal vector.

HDFs are represented as a percentage value following this procedure: initially expressed in Newtons, they are normalized with the volume of the left ventricle, subsequently divided by the fluid density and the gravitational acceleration *g* finally obtaining the following dimensionless form $\frac{F(t)}{\rho gV(t)}$.

The temporal profile of the longitudinal HDF ($F_{z}long$) was used to extract some key parameters (illustrated in Figure 2C) as described in [16]:

- LV longitudinal force (LVLF) as the mean amplitude of the longitudinal force throughout the cardiac cycle; since it includes both positive and negative values, the amplitude was computed as the root mean square of all values;
- LV systolic longitudinal force ($LV_{sys}LF$), calculated as the LVLF above but limited to the systolic phase only;
- LV systolic impulse ($LV_{sys}Im$) as the mean longitudinal force during the systolic propulsive phase, when the force is positive (directed from the LV cavity toward aorta); it is the area under the curve of the positive force profile during systole, normalized by the corresponding time interval;
- LV suction (LVs) as the mean longitudinal force during the period following propulsion while the force is negative; computed as $LV_{sys}Im$, it represents the force deceleration during the exiting flow, (with Aorta open and mitral valve closed) and the initial part of diastole (the effective suction when the mitral inflow accelerates, with Aorta closed and mitral valve open);
- LV diastolic longitudinal force ($LV_{dia}LF$), calculated as the LVLF above but limited to the diastolic phase only;
- LV diastolic impulse ($LV_{dia}Im$) as the mean longitudinal force during the diastolic propulsive phase, immediately after LVs (directed from the MV toward LV cavity); it is the area under the curve of the force profile during diastole, normalized by the corresponding time interval and computed as $LV_{sys}Im$.

### 2.6. Vorticity and Vortex Formation Time

The formation of the vortex and its orientation within the LV influence the correct course of the flow throughout the cardiac cycle until its expulsion [17]. The the average vorticity inside the ventricle, made dimensionless with the heartbeat period *T*, is evaluated by

(2) $$\bar{\omega }=\frac{T}{V}\int_{V}\omega \;dV,$$

where $\omega \left(x,t\right)=\left|\nabla \times v\right|$ is the modulus of the vorticity vector field.

The vortex formation time (VFT) is an important dimensionless parameter used for the evaluation of LV function [18]; this dimensionless parameter is computed as

(3) $$VFT=\int_{T_{E}}\frac{v_{MV}\left(t\right)}{d(t)}\;dt,$$

where $v_{MV}=\frac{Q_{MV}}{A_{eff}}$ is the mean velocity across the MV orifice, $d=\sqrt{\frac{4}{\pi }A_{eff}}$ the average MV orifice diameter, and $T_{E}$ is the diastolic E-wave period. This parameter measures the quality of the vortex formation process and optimal LV filling. Studies present in literature have shown that the optimal range is 3≤VFT≤4, although a value up to 5 is also considered acceptable [18]. High values are associated with the breakdown of the forming vortex and turbulence, while lower values correspond to suboptimal propulsion [19].

### 2.7. Tissue Dynamics

The dynamic properties of the cardiac tissue are primarily studied in terms of tissue deformation or strain. In our previous works [15], we provided a detailed description of strain analysis during cardiac deformation, addressing both the mathematical framework and clinical relevance. In this work, we present only a brief summary of the methodology.

Starting from an initial reference state at time t = 0, corresponding to the end of diastole, the simple definition for a *strain tensor* (known as the Lagrangian strain) is

(4) 𝘚t=U−I;

where U is the right stretch tensor that contains the information about the deformation occurred during the time interval (0,t) and I the identity matrix.

Cardiac contraction is modeled to begin at t=0, corresponding to an undeformed reference configuration taken at the end of diastole-the point at which LV volume is maximal- and proceeds until the end of systole, when the volume is minimal and strain eigenvalues become negative. LV deformation is quantified using global principal strain (GPS), the most negative eigenvalue of the strain tensor (4), and global secondary strain (GSS), the second eigenvalue. The principal direction of contraction is determined by the eigenvector corresponding to the GPS. Strain is evaluated both as a spatial distribution of GPS values and directions at the end of systole and as global (spatially-averaged) values of GPS and GSS over time throughout the cardiac cycle. For consistency with existing literature, global longitudinal strain (GLS) and global circumferential strain (GCS) were also computed.

### 2.8. Statistical Analysis

Data are expressed as means ± standard deviation for continuous variables. Normal distribution of continuous variables was checked using skewness and kurtosis. For the current analysis, group means were compared using the unpaired *t*-test between Control and Children at *Rest* phase cases. Through linear regression analysis, we calculated the correlations between continuous variables. Statistical significance is a two-sided level of 0.05. Data were analyzed using the Statistics and Machine Learning Toolbox (Matlab R2024b, MathWorks, Natick, MA, USA).

## 3. Results

The control group, when compared with the *Rest* phase of the exercise cohort, showed close similarity in both geometry (ventricular volumes) and contraction properties (EF and strain). This confirms the suitability of the control data for comparison with subsequent phases of the exercise protocol. As the control cases are visually indistinguishable from the *Rest* phase of the exercise group, their graphs are not shown.

**Figure 3:**
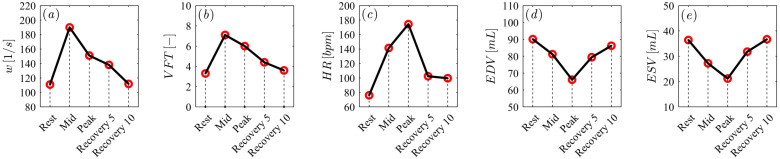
(**a**) Vorticity diastolic-peak values, (**b**) VFT values, (**c**) Heart Rate values, (**d**) EDV values and (**e**) ESV values for all subjects measured at the five time points (*Rest*, *Mid*, *Peak*, *Recovery 5* and *Recovery 10*).

During exercise, the ventricle must pump a greater volume of blood per minute to meet increased physiological demand. This is achieved by increasing heart rate and increasing muscle contractility, resulting in a reduction in ventricular volume with increasing exercise (Figure 3c–e). Several changes in intraventricular vorticity also occur, a very important parameter that provides clinically important information on cardiac health during LV diastolic filling. Vortex formation during fluid propagation from the left atrium to the left ventricle is important for efficient fluid transport. Quantifying VFT can therefore help assess and understand disease and physio-pathological processes. Figure 3a,b show a significant increase in vorticity (computed with Equation (2)) and VFT (computed with Equation (3)) during the *Mid* phase, consistent with the sudden effort that occurs from the *Rest* phase to the next one.

**Figure 4:**
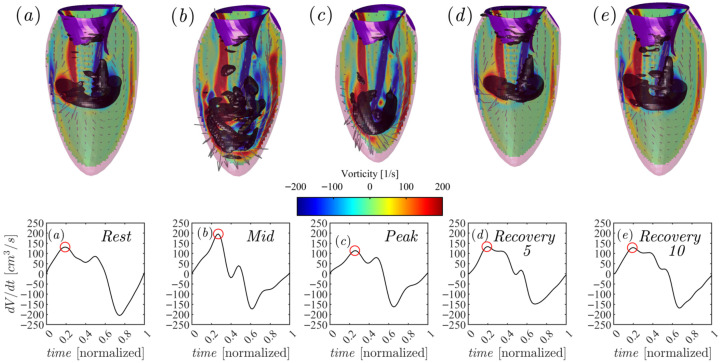
Diastolic flow field at the peak of the E wave for time points (**a**) *Rest*, (**b**) *Mid,* (**c**) *Peak*, (**d**) *Recovery 5* and (**e**) *Recovery 10*, respectively.

In Figure 4 we show the vortex formation at the peak of the E-wave in all phases of the exercise. In the *Rest* phase, the vortex is asymmetric and regular, in line with existing literature both measured in vivo with 3D phase-contrast MRI (known as 4D flow MRI) [20] and with numerical simulation methods [21].

As can be observed, as HR increases, diastolic time decreases (Figure 4), as shown in Table 1. This physiological variation is reflected in the dV/dt ratio in Figure 4, with the variation in the slope of the curve in the ventricular filling phase influencing MV opening [13]. In particular, during the *Mid* and *Peak* phases, the vortex ring reaches the peak of the E-wave close to the rupture, while the high VFT ($VFT_{Mid}$∼ 7 vs. $VFT_{Peak}$∼ 6) indicates instability in fluid dynamic transport, difficulty in forming an adequate vortex resulting in high vorticity during early diastole. In the Recovery phases, however, both the vortex ring and the VFT return to being similar to the Rest case. In the *Recovery* phases, however, while the vortex ring immediately returns to the similar state of the Rest case, the VFT requires 10 min to return to the range defined as physiological by the literature [18]. The study of vortices and VFTs in stress echocardiography proves to be important markers for the analysis and identification of morphological remodeling of the heart in athletes and can also contribute to and define the effects of training intensity and energy expenditure [22].

The atrial contribution varies during the different phases of exercise. The E/A value (dV/dt curves of Figure 4), an indicator of left ventricular function, is greater than 2 in the *Mid* phase (E/A = 6.3), with a greater contribution from the E-wave than from the atrium (A-wave). In the *Peak* phase, the atrial/ventricular contribution is uniform (E/A = 1.5). In the *Recovery* phases, the two waves almost seem to merge, significantly reducing diastasis and also the E/A ratio compared to the *Rest* phase (1.1 vs. 1.2 vs. 1.6, respectively for *Recovery 5*, *Recovery 10* and *Rest*). The systolic phase during exercise (dV/dt curves of Figure 4) shows a classic ejection phase in the *Rest* cases, and a steeper one immediately after the isovolumetric contraction in the *Mid* and *Peak* cases. In the *Recovery* phases, however, a positive peak is observed in the isovolumetric contraction phase and a subsequent ejection similar to the *Rest* case.

Several scientific articles [4,22,23] highlight the importance of vortex analysis and VFT for studying cardiac remodeling. In most cases, these analyses have been performed only during *Rest* and *Recovery* phases due to the difficulty of capturing optimal 2D echocardiographic images during the *Mid* and *Peak* phases, leaving a gap in what happens between Rest and Recovery.

**Figure 5:**
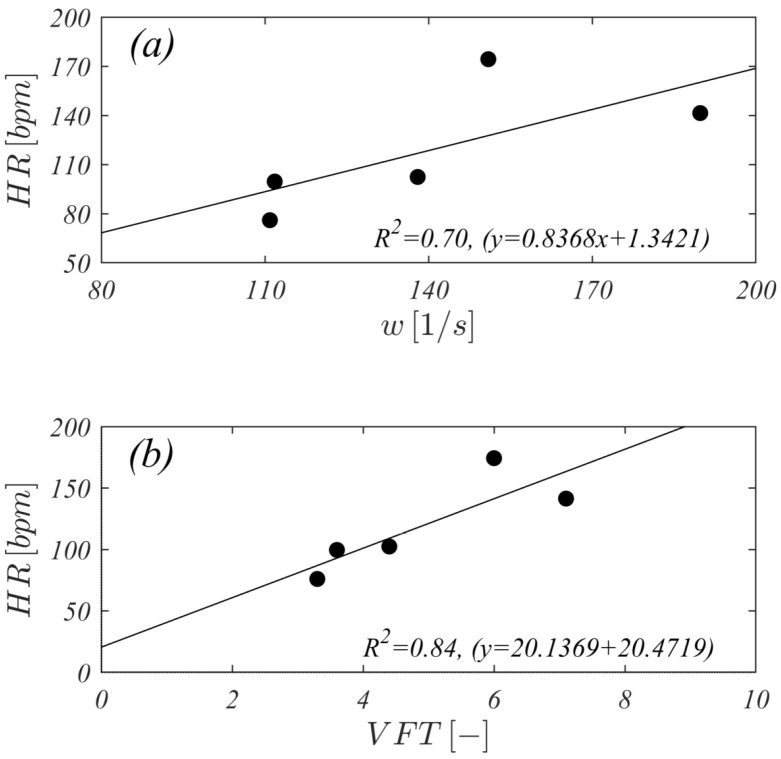
Relations of HR (vertical axis) with (horizontal axis axes): (**a**) diastolic vorticity peak and (**b**) VFT.

Through this study we tried to extend the knowledge in all phases of physical exercise, demonstrating the clear correlations among vorticity, HR and VFT as shown in Figure 5. The $R^{2}$ graphs (Figure 5) show an acceptable correlation despite being average values calculated directly from the results of numerical simulations.

**Figure 6:**
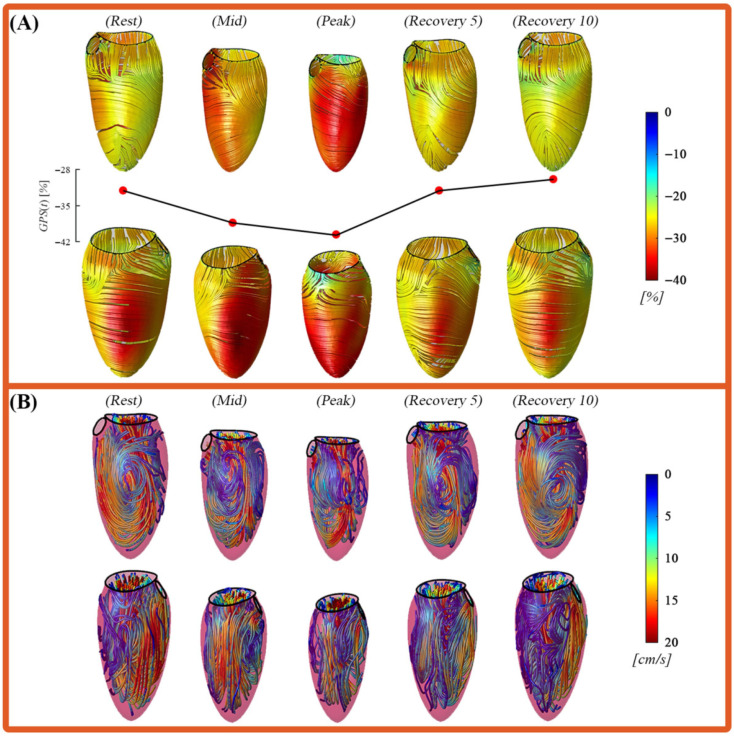
(**A**) Color map of the first principal strain (most-negative eigenvalue of the strain tensor) at the end of systolic contraction, each one shown in two opposite facing views (anterior and posterior). Colors are drawn over the strain-lines (tangent to the corresponding eigenvector). (**B**) Streamlines of the steady-streaming (heartbeat-average), each one shown in two opposite facing views (anterior and posterior). Streamlines are colored by velocity magnitude. All shown for phases (*Rest*), (*Mid*), (*Peak*), (*Recovery 5*) and (*Recovery 10*) respectively.

Furthermore, the increase in contraction is reflected in a progressively more negative GPS as exercise intensity increases. Additionally, this enhanced contraction leads to a more uniform spatial distribution of GPS (computed with Equation (4)) across the ventricular wall. Figure 6A illustrates the spatial distribution of GPS across the five phases of the exercise protocol for the group-averaged subject. The color map overlays *strain-lines*, each tangent to the corresponding eigenvector direction, providing a visual representation of both strain magnitude and orientation.

At the *Rest* phase, LV strain directions exhibit regional variation, ranging from circumferential to right-handed or left-handed helical orientations throughout the ventricular wall. This pattern reflects the anatomical arrangement of myocardial fibers, which transition from a right-handed helix in the inner layer (sub-endocardium), to circumferential orientation in the mid-myocardium, and to a left-handed (counter-rotating) helix in the outer layer (sub-epicardium) [24]. As workload increases during the *Mid* and *Peak*, the direction of deformation tends to shift toward a more circumferential alignment. This shift results from the coordinated activation of multiple myocardial layers with varying strain-lines directions, allowing the heart to recruit contractile forces across the entire wall thickness to achieve higher performance. In functional terms, the simultaneous contraction along multiple directions produces a strain tensor whose GPS aligns more closely with the circumferential direction, while the GSS becomes more aligned with the longitudinal direction.

**Figure 7:**
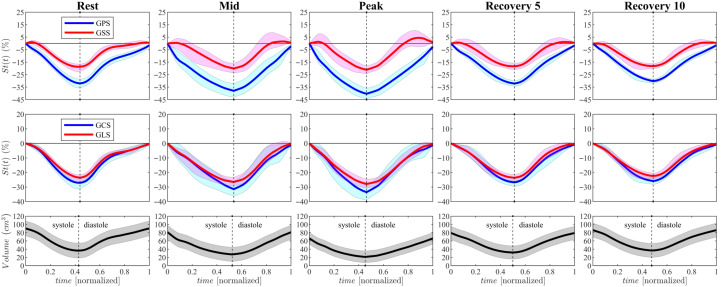
Global value of Principal Strain (GPS, blue curves) and Secondary Strain (GSS, red) for all subjects measured at each time points (Rest, Mid, Peak, Recovery 5, Recovery 10); Volume curves are reported below for reference; the line reports the average value and the surrounding shadow is ±the standard deviation.

This picture can be reinforced by the analysis of the time course of GPS and GSS (computed with Equation (4)) shown in Figure 7: immediately at the onset of contraction the former presents a sharp decrease, such a strong contraction in the dominant direction provokes an initial stretch in the perpendicular direction and GSS increase to a positive peak; soon afterwards, all fibers become active for the contraction, both GPS and GSS decrease and reach, at end systole, their stronger exercise-induced value. Finally, in the *Recovery* phase, it is interesting to note that, both after 5 and 10 min, this normal degree of contraction is reached with a different contraction pattern that testifies to the persistent modification of the left ventricular function induced by exercise, despite the global contractile values returning similar to those at *Rest*.

This trend is supported by the data in Table 1, which shows a reduction in ventricular length corresponding to increased heart rate (HR) as exercise intensity rises. These changes are consistent with the observed strain distributions described earlier and correlate with variations in intraventricular flow dynamics, as illustrated in Figure 6B and discussed in the following section.

In the *Rest* phase, the intraventricular flow exhibits the classic rotational pattern typically observed in healthy individuals [15]. However, during the *Mid* phase, triggered by the instantaneous effort given by the first phase of physical exercise, the flow pattern undergoes directional modifications. These are primarily due to increased flow impacts against the anterior and inferior ventricular segments during the rotational phase, which in turn alter the trajectory of the flow toward the aortic outflow tract. In the *Peak* phase, the ventricular motion becomes notably faster and more forceful, as previously indicated by the strain and flow parameters. This results in a higher flow intensity, with more pronounced impacts in the anterior and inferior regions. The classic rotational flow pattern becomes disrupted, showing a shift toward a more direct and immediate orientation toward the outflow tract, facilitating rapid ejection. Notably, the apical region maintains a consistent flow pattern across all five exercise phases.

Finally, during the *Recovery* phases, LV gradually returns toward its baseline functional state. The flow pattern begins to stabilize, becoming more regular and increasingly similar, but not identical, to that observed during the *Rest* phase. As shown in Table 1 and Figure 6, strain values during the *Recovery 10* phase remain lower than the *Rest*, indicating a state of enhanced ventricular relaxation. This likely reflects residual cardiac fatigue from the prior exertion, as evidenced by the persistent predominance of GPS/GCS over GSS/GLS (computed with Equation (4)). These results confirm how a small change during the diastolic phase can significantly influence the rest of the cardiac cycle.

**Figure 8:**
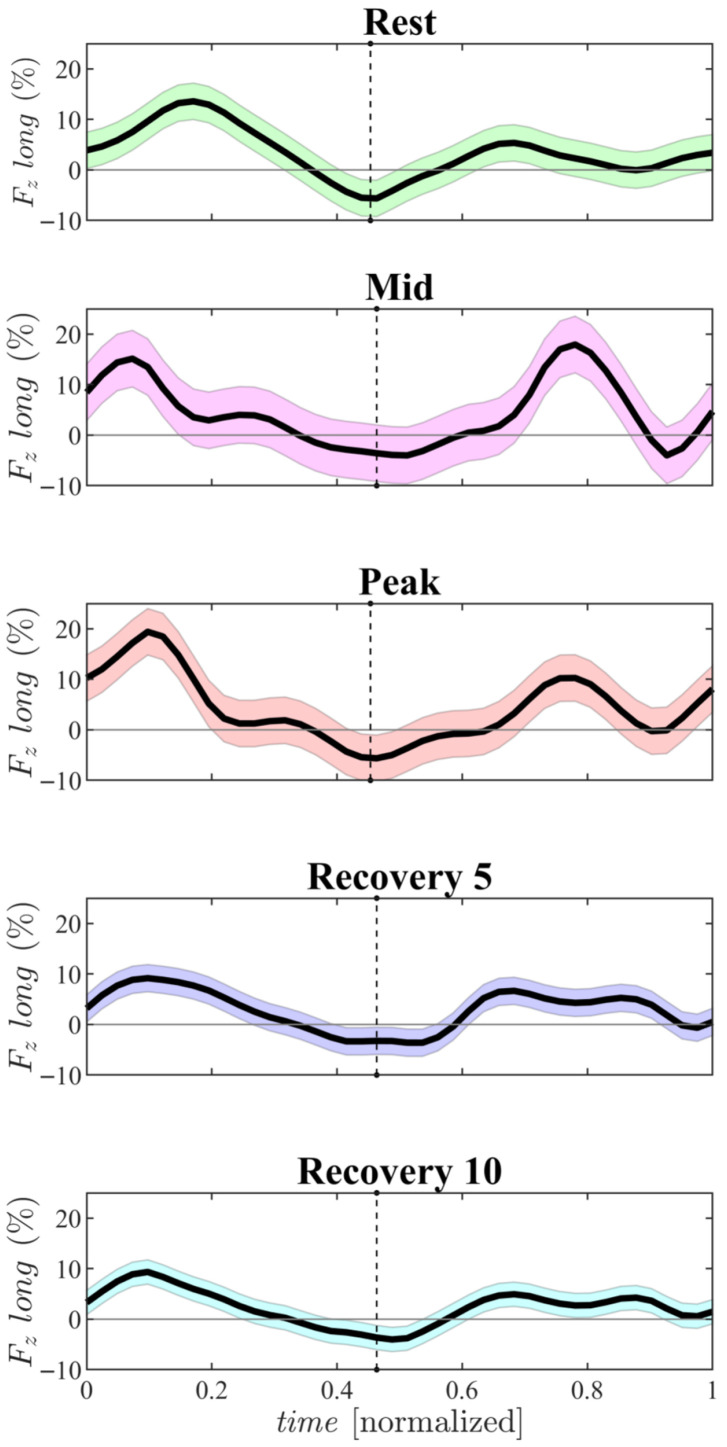
Time course of longitudinal components of the dimensionless hemodynamic force vector. Each curve represents the average computed for respective time points (Rest, Mid, Peak, Recovery 5, Recovery 10); the line reports the average value and the surrounding shadow is ±the standard deviation. The black dashed line indicate the separation between systolic (left side) and diastolic (right side) period.

Further insight into the physiological response to exercise is provided by the HDFs (computed with Equation (1)), illustrated in Figure 8. At the *Rest* phase HDFs display a normal distribution, with magnitudes (shown Table 1) consistent with previously reported values in the literature [15]. During the *Mid* phase, there is a sharp increase in both the systolic peak and a diastolic predominant- indicative of an early and acute elevation in HR. In the *Peak* phase, the systolic force increases further, while the diastolic peak reduces slightly (though still remains elevated compared to *Rest*) resulting in a more uniform force distribution throughout the cardiac cycle. These elevated values reflect the redistribution of cardiac workload and altered flow dynamics under physical stress, a phenomenon supported by similar findings in the literature [16]. During the early recovery period (*Recovery 5*), HDF values begin to return toward baseline, yet remain distinct from Rest—underscoring the physiological importance of a gradual cool-down following intense exercise. By the *Recovery 10* phase, this relaxation phase continues, with HDF values falling below those observed at *Rest*. This is a novel observation, suggesting a prolonged recovery response in individuals following high-intensity physical exercise.

## 4. Limitations

This study has several limitations. It is a preliminary investigation with a small patient sample. Another limitation, as discussed in Section 2.2, is the low number of frames acquired as exercise intensity increases, due to the limited ability of 3D echocardiography to track the progressively higher velocities of the endocardial wall. The computational limitations have been discussed in our previous works [13,15], and are therefore not reported here.

## 5. Conclusions

The combined assessment of LV tissue and intraventricular fluid dynamics coupling during physical exercise allows a deeper understanding of the mechanical adaptations induced by physiological stress. Our results show evidence of modifications in the cardiac deformation pattern in healthy children during physical exercise. These modifications are characterized by increased myocardial deformation along all directions, with the principal direction of contraction shifting toward a predominantly circumferential orientation. This shift is accompanied by a corresponding reorientation of intraventricular flow, particularly at peak exercise phase, where flow impacts become more directionally focused toward the outflow tract, reflecting optimized ejection dynamics. This observation is further supported by the analysis of vorticity and VFT which confirm that such ventricular variations under stress coincide with a variation in the formation and distribution of the vortex and therefore of the VFT, important markers for the evaluation of cardiac performance in correlation with tissue modification under stress. These changes are also reflected in the temporal evolution of HDFs throughout the cardiac cycle. Exercise induces a prompt and generalized increase in HDF magnitude [16,25], which correlates closely with rising HR and strain values. During the recovery phases, these forces rapidly decline, yet do not fully return to baseline, highlighting a lasting effect of acute exertion on ventricular mechanics.

## 6. Discussion

The interpretation of strain data suggests that physical exercise alters the activation of myocardial fibers across the full thickness of the ventricular wall throughout the different phases of exertion. This observation aligns with the increased myocardial thickness typically seen in athletes’ hearts, reinforcing the idea that physiological hypertrophy is a reversible and beneficial response, clearly distinct from pathological hypertrophy. These adaptations are also reflected in the temporal evolution of HDFs across the cardiac cycle. Exercise triggers an immediate and widespread increase in HDF magnitude [16], closely tracking the rise in heart rate and strain values. During recovery, these forces decrease rapidly but do not fully return to baseline, suggesting that even short periods of exertion leave a measurable, lingering effect on ventricular mechanics. Collectively, these findings provide mechanical support for exercise-based therapeutic strategies, showing how physical activity enhances myocardial efficiency by recruiting fiber layers throughout the ventricular wall [26]. Additionally, exercise in children under stress shows a pronounced association with diastolic dysfunction. As illustrated by the volume curves in Figure 7, the relaxation phases under exertion appear prolonged and incomplete. Heart rate increases as a compensatory mechanism, shortening diastole and counterbalancing the reduced filling time—features typically associated with diastolic dysfunction. During *Rest*- and *Peak*-exercise, an E/A ratio greater than 2 resembles patterns observed in grade II–III diastolic dysfunction, characterized by severe ventricular filling and limited LV relaxation capacity. These results may provide an early indication of pathological conditions such as ventricular hypertrophy and diastolic dysfunction, which are often linked to common risk factors including hypertension, aging, obesity, diabetes mellitus, and the development of atrial fibrillation (AF). Clinical studies on diastolic dysfunction and non-valvular AF in individuals without structural heart disease suggest that elevated filling pressures and left atrial enlargement may play central roles in disease progression. Increased atrial afterload, stretching of atrial myocytes, and atrial wall stress are key mechanisms through which diastolic dysfunction may increase AF risk—mechanisms that align well with the patterns observed in our data. These correlations offer a potential pathway for the early detection of diastolic dysfunction [27]. Moreover, diastolic dysfunction has been independently associated with abnormal heart-rate recovery after symptom-limited exercise [28]. The structural changes observed in vortex formation, together with the substantial rise in VFT during exertion, highlight an important functional marker that has remained largely unexplored in the study of LV mechanics during physical activity. From a methodological perspective, this preliminary study demonstrates the feasibility and promise of integrating 3D echocardiography–based tissue–flow coupling analysis to detect early alterations in cardiac function. Continued refinement and validation of this approach may offer valuable insights into the progression of cardiac remodeling during recovery, athletic training, or early disease, thereby expanding the clinical applicability of advanced echocardiographic modeling in both research and clinical practice.

## Figures and Tables

**Table 1:** Population characteristics and hemodynamic changes in control subjects and in enrolled children during exercise phases. HR = Heart Rate, EDV = End Diastolic Volume, ESV = End Systolic Volume, SV = Stroke Volume, EF = Ejection Fraction, GPS = Global Principal Strain, GSS = Global Secondary Strain, GLS = Global Longitudinal Strain, GCS = Global Circumferential Strain, LVLF = LV Longitudinal Force, ${\mathrm{LV}}_{\mathrm{dia}}\mathrm{LF}$ = LV systolic Longitudinal Force, ${\mathrm{LV}}_{s}$ = LV suction, ${\mathrm{LV}}_{\mathrm{dia}}\mathrm{LF}$ = LV diastolic Longitudinal Force, LVdiaIm = LV diastolic Impulse, M = Male, F = Female. The values, at differences of the Gender, are reported as mean ± SD. * *p*-Value Unpaired *t*-test between Control and Children at *Rest* phase.

	Controls	Rest	Mid	Peak	Recovery 5	Recovery 10	*p* *
HR [bpm]	77±9	76±11	141±19	174±10	102±11	100±12	0.001
EDV [mL]	89±21	90±24	81±31	66±19	79±17	86±19	0.832
ESV [mL]	35±13	36±10	27±8	21±6.4	32±6	37±8	0.344
SV [mL]	54±16	54±15	54±26	45±14	48±12	49±12	0.764
EF [%]	61±4	59±4	65±6	68±4	59±2	57±2	0.002
GPS [%]	−33±3	−32±3	−38±6	−40±4	−32±3	30±2	0.001
GSS [%]	−18±2	−19±2	−20±4	−21±3	−18±2	−18±2	0.129
GLS [%]	−23±2	−24±2	−27±3	−28±3	−24±2	−22±2	0.196
GCS [%]	−28±3	−27±4	−31±5	−34±5	−27±3	−26±2	0.003
LVLF [%]	5±4	5±3	6±2	6±1	4±1	3±2	<0.001
${\mathrm{LV}}_{\mathrm{sys}}\mathrm{LF}$ [%]	7±3	7±4	6±2	7±2	5±2	4±2	0.004
LVsysIm [%]	8±3	8±4	7±3	9±4	6±2	5±2	0.004
${\mathrm{LV}}_{s}$ [%]	3±2	3±2	2±2	3±1	2±1	2±1	<0.001
${\mathrm{LV}}_{\mathrm{dia}}\mathrm{LF}$ [%]	3±2	3±2	6±1	4±1	4±1	3±1	<0.001
LVdiaIm [%]	7±3	7±4	7±3	10±3	5±2	4±2	<0.001

## Data Availability

The raw data supporting the conclusions of this article will be made available by the authors, without undue reservation.
